# Noninvasive mechanical ventilation assistance in amyotrophic lateral sclerosis: a systematic review

**DOI:** 10.1590/1516-3180.2022.0470.R1.100423

**Published:** 2023-07-07

**Authors:** Carolina da Cunha-Correia, Mylana Dandara Pereira Gama, Pedro Nogueira Fontana, Francisca Goreth Malheiro Moraes Fantini, Gilmar Fernandes Prado, Mário Emílio Teixeira Dourado, Paulo Adriano Schwingel

**Affiliations:** I PhD. Neurologist and Professor, Department of Neurology, Hospital Universitário Oswaldo Cruz (HUOC), Universidade de Pernambuco (UPE), Recife (PE), Brazil.; II MD. Resident Physician, Department of Neurology, Hospital Universitário Oswaldo Cruz (HUOC), Universidade de Pernambuco (UPE), Recife (PE), Brazil.; III MSc. Neurologist, Department of Neurology, Hospital Universitário Oswaldo Cruz (HUOC), Universidade de Pernambuco (UPE), Recife (PE), Brazil.; IV MD. Neurologist and Neurophysiologist, Santa Casa de Misericórdia de Fernandópolis (SCF), Fernandópolis (SP), Brazil.; V PhD. Neurologist and Professor, Department of Medicine, Escola Paulista de Medicina (EPM), Universidade Federal de São Paulo (UNIFESP), São Paulo (SP), Brazil.; VI PhD. Neurologist and Professor, Department of Integrated Medicine, Centro de Ciências da Saúde (CCS), Universidade Federal do Rio Grande do Norte (UFRN), Natal (RN), Brazil.; VII PhD. Sports Physiologist and Associate Professor, Human Performance Research Laboratory, Universidade de Pernambuco (UPE), Petrolina (PE), Brazil.

**Keywords:** Amyotrophic lateral sclerosis, Respiratory insufficiency, Noninvasive ventilation, Survival, Quality of life, Motor neuron diseases, Respiratory failure, Hypoventilations, Artificial respiration, Health system, Health, attitude to

## Abstract

**BACKGROUND:**

Respiratory failure is the most common cause of death in patients with amyotrophic lateral sclerosis (ALS), and morbidity is related to poor quality of life (QOL). Non-invasive ventilation (NIV) may be associated with prolonged survival and QOL in patients with ALS.

**OBJECTIVES:**

To assess whether NIV is effective and safe for patients with ALS in terms of survival and QOL, alerting the health system.

**DESIGN AND SETTING:**

Systematic review was conducted in accordance with Preferred Reporting Items for Systematic Reviews and Meta-Analyses reporting standards using population, intervention, comparison, and outcome strategies.

**METHODS:**

The Cochrane Library, CENTRAL, MEDLINE, LILACS, EMBASE, and CRD databases were searched based on the eligibility criteria for all types of studies on NIV use in patients with ALS published up to January 2022. Data were extracted from the included studies, and the findings were presented using a narrative synthesis.

**RESULTS:**

Of the 120 papers identified, only 14 were related to systematic reviews. After thorough reading, only one meta-analysis was considered eligible. In the second stage, 248 studies were included; however, only one systematic review was included. The results demonstrated that NIV provided relief from the symptoms of chronic hypoventilation, increased survival, and improved QOL compared to standard care. These results varied according to clinical phenotype.

**CONCLUSIONS:**

NIV in patients with ALS improves the outcome and can delay the indication for tracheostomy, reducing expenditure on hospitalization and occupancy of intensive care unit beds.

**SYSTEMATIC REVIEW REGISTRATION:**

PROSPERO database: CRD42021279910 — https://www.crd.york.ac.uk/prospero/display_record.php?RecordID=279910.

## INTRODUCTION

Amyotrophic lateral sclerosis (ALS) is a rare, progressive neurodegenerative disease characterized by the irreversible loss of motor neurons. This leads to generalized paralysis and respiratory insufficiency, mainly due to diaphragmatic weakness, which is the main cause of death from this disease.^1^

Episodes of acute decompensation frequently occur during simple upper airway infections. This is facilitated by the inability of individuals to eliminate secretions and weakness of their oropharyngeal musculature, with consequent bronchoaspiration.^2,3^

Epidemiological data indicate that the onset of the disease can affect younger individuals, especially in genetic forms.^4^ This aspect also calls attention to the need for noninvasive ventilator assistance (NIV) with the aid of two-level volumetric ventilators to prolong survival and increase the involvement of respiratory specialists in this assistance.^5–7^

Considering that, in some countries, NIV prescription is still influenced by insurance and financial constraints, and that some publications diverge with regard to timing and prognostic factors, the current context reinforces the need for a systematic review on the subject.

## OBJECTIVE

This systematic review utilized the Population, Intervention, Comparison, Outcomes (PICO)^8^ strategy to focus on the effectiveness and safety of NIV and assess its impact on the survival and quality of life of patients with ALS and respiratory failure.

## METHODS

### Design and setting

The review protocol was registered with the International Prospective Register of Systematic Reviews database (www.crd.york.ac.uk/prospero/; registration number CRD42021279910) and was conducted in accordance with the Preferred Reporting Items for Systematic Reviews and Meta-Analyses (PRISMA) reporting standards^9^ using the PICO strategy for research question construction and evidence search.^8,10^

### Research strategy

Electronic databases were searched, without language or time restrictions, for relevant studies published until January 2022: Cochrane Library, Cochrane Central Register of Controlled Trials (CENTRAL), MEDLINE via PubMed, Literatura Latino-Americana e do Caribe em Ciências da Saúde (LILACS), Excerpta Medica Database (EMBASE), Center for Reviews and Dissemination (CRD), Cumulative Index to Nursing and Allied Health Literature (CINAHL) Plus, and the Allied and Complementary Medicine Database.

Supplementary research was conducted on the websites of health technology assessment agencies, correlated institutions, and their databases. Electronic searches were complemented by manual searches of the reference lists of the included studies and grey literature searches.

Specific descriptors, keywords, Embase subject headings (Emtree), and Medical Subject Headings (MeSH) for each database were used to construct the search strategies. Supplementary material available at https://doi.org/10.6084/m9.figshare.22321504 presents the search strategy adopted. Systematic reviews of randomized clinical trials with or without meta-analyses, and individual randomized clinical trials on the use of NIV in patients with ALS were sought. Systematic reviews of good methodological quality with meta-analyses were prioritized because of their higher levels of evidence. If no such reviews existed, a search for individual studies was planned.

The search strategy was created using the PICO strategy.^10^ The patients (P) used in the search strategy were those with ALS and an indication for ventilatory assistance. A rapid initial investigation was conducted to create an intervention section for the search strategy. Intervention (I) was the indication for NIV. Comparison (C) was defined as a standard treatment that did not involve NIV or other comparators. Outcomes (O) are related to quality of care, including survival, quality of life, and other clinical outcomes.

For this review, the following studies were excluded: duplicates, non-comparative studies, comparative studies with a retrospective design, and studies published only in an abstract format or the like, without complete data that would make it possible to assess the methods.

The two reviewers searched the databases using a previously defined strategy. Based on these criteria, they selected studies for inclusion in this review. In the event of a lack of consensus between the two reviewers, a third reviewer was consulted regarding eligibility and was responsible for making the final decision. The included studies were evaluated for level of evidence using the Oxford Centre of Evidence-Based Medicine Levels of Evidence.^11^

This review was conducted in accordance with Preferred Reporting Items for Systematic Reviews and Meta-Analyses recommendations^9^ and is presented using narrative synthesis.

## RESULTS

### Search results

**Figure 1:**
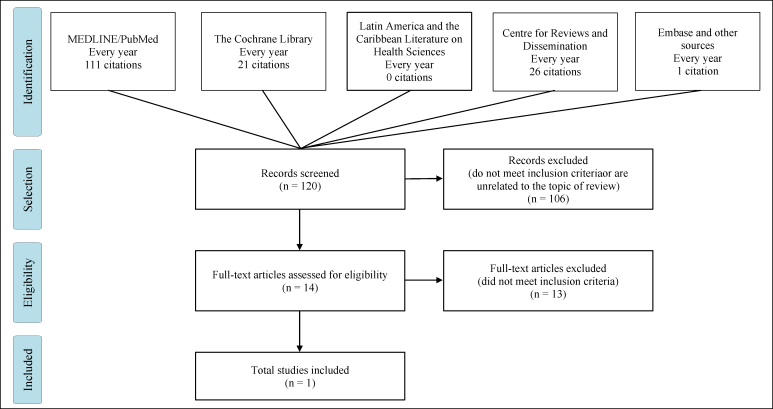
Preferred reporting itens for systematic reviews and meta-analyses.

The articles were selected in stages. First, systematic reviews with or without meta-analyses were selected, given their higher quality regarding the hierarchy of evidence. Through a search of the databases for systematic reviews, 120 non-duplicated titles were identified. Two reviewers applied the eligibility criteria and initially selected 14 articles for full reading that were potentially related to systematic reviews. Among these, one systematic review and meta-analysis by the Cochrane Collaboration was considered eligible (Figure 1). Two systematic reviews with adequate search and selection methods were excluded after reading them completely because they included non-comparative cohort studies with prospective or retrospective designs.

**Figure 2:**
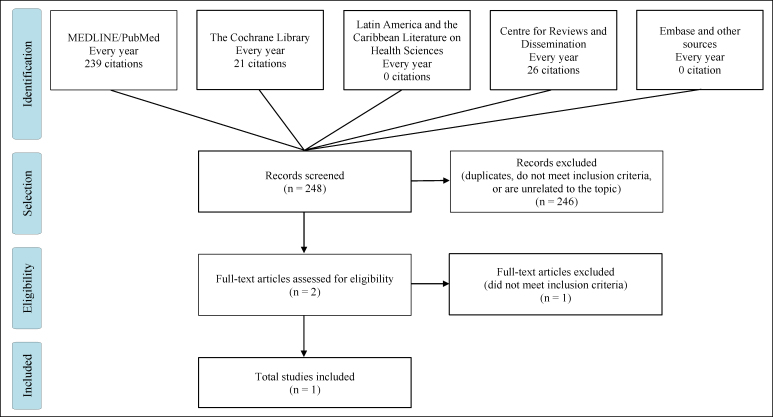
Flow diagram showing study selection method (efficacy and safety in prospective comparative studies or randomized clinical trials).

In the second stage, the individual studies were screened and selected according to the strategies described above. Of the 248 registered articles, two were selected for full reading. Only two were selected and included in this review (Figure 2).

### A systematic review with meta-analysis produced through the Cochrane Collaboration

A systematic review was conducted in accordance with the guidelines of the Cochrane Collaboration, in which they sought to evaluate the effects of NIV and ventilation assisted through tracheostomy (VAT) on survival, functional parameters and quality of life among patients with ALS. Furthermore, they sought to evaluate the safety of these technologies.^5^

Searches were conducted up to January 2022 using the following databases: Cochrane Library, CENTRAL, MEDLINE, EMBASE, LILACS, CRD, CINAHL Plus, and Allied and Complementary Medicine Database. The eligibility criteria for the review were: the studies needed to be randomized clinical trials (RCTs) or quasi-randomized studies involving NIV or VAT among participants with a clinical diagnosis of ALS, independent of the outcomes reported. Comparisons between the best standard of treatment and the absence of treatment were also considered eligible.

Initially, two RCTs involving 54 participants with ALS receiving NIV were included. One of these studies (n = 13) compared the early and late use of NIV and presented incomplete data. Missing data were not made available by the original authors of the article even after contacting the Cochrane reviewers. Therefore, this study was excluded from the analysis.

In the second study (n = 41), NIV was compared with usual care. This study was eligible for inclusion in this review. The Cochrane authors assessed the risk of bias in this study and noted that the lack of blinding constituted a risk from the point of view of the outcomes reported by the participants and doctors. However, overall, this was a well-designed and well-conducted study, providing evidence of moderate quality that the overall median survival was significantly different between the group treated with NIV and the group that received usual care. The median survival time in the NIV group was 48 days longer (219 versus 171 days; 95% confidence interval, 12–91 days; P = 0.0062). This survival benefit was accompanied by an improvement in the quality of life. In the subgroup analyses, the median survival in the subgroup with normal or moderately impaired bulbar function (20 patients) was 205 days greater (216 versus 11 days; P = 0.0059), and the quality of life was better than that in the group receiving usual care. In the subgroup with poor bulbar function (21 participants), NIV did not prolong survival or improve quality of life, although there was a significant improvement in the domain of symptoms of the Sleep Apnea Quality of Life Index (SAQLI). None of the studies reported any data on safety or adverse events.

Table 1 demonstrates that there was evidence of moderate quality from a single RCT comparing the use of NIV with the best usual care among the 41 patients, indicating that NIV significantly prolonged the survival of patients with ALS. Additionally, there was evidence that the quality of life was maintained at better levels for a longer time among patients receiving NIV in comparison with those who received usual care without NIV. However, the results regarding this outcome might be due to the lack of blinding between the groups. Moreover, survival and some measurements of quality of life were significantly better in the subgroups with better bulbar function than in the subgroup with severe bulbar impairment. Adverse events related to NIV or the comparators were not evaluated or reported; thus, no information was obtained from the RCT.

**Table 1:** Summary of the main results from Radunovic et al.^5^

Outcomes	Comparative risks (95% confidence interval)		Number of participants	Quality of evidence (GRADE)
	Assumed risk	Corresponding risk		
	Usual care	Noninvasive ventilation		
**Survival**	**All participants** Median survival 171 days	**All participants** Median survival 48 days longer (12 to 19)	41 (one study)	Moderate
	**Participants with better bulbar function** Median survival 11 days	**Participants with better bulbar function** Median survival 205 days longer (CI not reported)		
	**Participants with poor bulbar function** Median survival 261 days	**Participants with poor bulbar function** Median survival 39 days shorter (statistically non-significant)		
**Quality of life (SF-36 Mental component summary)**	**All participants** Median length of time for which the score remained more than 75% above the baseline was 99 days	**All participants** Median length of time for which the score remained more than 75% above the baseline was 69 days longer (45 to 667)	41 (one study)	Low
	**Participants with better bulbar function** Median length of time for which the score remained more than 75% above the baseline was 4 days	**Participants with better bulbar function** Median length of time for which the score remained more than 75% above the baseline was 195 days longer (P = 0.001; CI not reported)		
	**Participants with poor bulbar function** Median length of time for which the score remained more than 75% above the baseline was 164 days	**Participants with poor bulbar function** Median length of time for which the score remained more than 75% above the baseline was 37 days shorter (P = 0.64; CI not reported)		
**Quality of life (SF-36 Physical component summary)**	**All participants** Median length of time for which the score remained more than 75% above the baseline was 81 days	**All participants** Median length of time for which the score remained more than 75% above the baseline was 69 days longer (P = 0.004)	41 (one study)	Low
	**Participants with better bulbar function** Median length of time for which the score remained more than 75% above the baseline was 4 days	**Participants with better bulbar function** Median length of time for which the score remained more than 75% above the baseline was 175 days longer (P < 0.001)		
	**Participants with poor bulbar function** Median length of time for which the score remained more than 75% above the baseline was 132 days	**Participants with poor bulbar function** Median length of time for which the score remained more than 75% above the baseline was 18 days longer (P = 0.88)		
**Sleep Apnea Quality of Life Index (SAQLI)**	**All participants** Median length of time for which the score remained more than 75% above the baseline was 99 days	**All participants** Median length of time for which the score remained more than 75% above the baseline was 74 days longer (P = 0.031)	41 (one study)	Low
	**Participants with better bulbar function** Median length of time for which the score remained more than 75% above the baseline was 4 days	**Participants with better bulbar function** Median length of time for which the score remained more than 75% above the baseline was 195 days longer (P < 0.001)		
	**Participants with poor bulbar function** Median length of time for which the score remained more than 75% above the baseline was 132 days	**Participants with poor bulbar function** Median length of time for which the score remained more than 75% above the baseline was 29 days shorter (P = 0.77)		

SF-36: Medical Outcomes Study 36-item Short-Form Health Survey; GRADE = Grading of Recommendations Assessment, Development and Evaluation.

### Comparative observational study

Berlowitz et al.^12^ conducted a study covering the period between 1991 and 2011 to determine the effects of NIV on survival and pulmonary function among patients with ALS of all phenotypes. They included 1198 patients from the Bethlehem Hospital database. Of them, 929 (77.5%) met the eligibility criteria and were included in the analysis. The phenotypic distribution was as follows: bulbar ALS, n = 312 (33.5%); cervical ALS, n = 240 (25.8%); lumbar ALS, n = 295 (31.7%); flail arms, n = 62 (6.6%); and flail legs, n = 21 (2.2%). As the samples of the flail arm and leg phenotypes were small, and their patterns of disease progression and survival were similar, they were considered together as the flail limb group (n = 83; 8.9%).

Univariate comparisons were made between baseline characteristics and survival analyses, with adjustments for age at disease onset, sex, use of riluzol, and use of percutaneous endoscopic gastrostomy. In addition, a mixed-model analysis was used to assess the rate of decline in respiratory function (forced vital capacity, forced expiratory volume in 1 second, maximal inspiratory pressure, maximal expiratory pressure, and sniff nasal inspiratory pressure) before and after initiation of NIV. This model enabled analysis involving “before and after” comparison among patients using NIV, in which the main parameter of interest was the interaction between the use of NIV and the time elapsed since the start of NIV use. As this analysis did not make a comparison with any group of patients who did not receive NIV, these results were not considered eligible and were not included as an outcome of interest in the present review.

As shown in Table 2, with regard to survival, Cox univariate regression showed that among the individuals using NIV, survival was almost 40% longer for all phenotypes of ALS (hazard ratio [HR] = 0.61). The positive effect of NIV on survival was maintained after adjusting for model for age at symptom onset, sex, use of riluzol, and use of gastrostomy (HR = 0.72). Tracheostomy-free survival starting from the time of symptom onset was 28 months among patients treated with NIV compared to 15 months among patients who did not receive NIV. Among the patients with bulbar ALS, NIV significantly increased survival by 19 months (univariate HR = 0.50; multivariate HR = 0.59). The survival advantage observed among patients with onset of bulbar disease was confirmed by a sensitivity analysis conducted by the authors using a paired cohort model.

**Table 2:** Analysis on Cox univariate and multivariate survival

Variables	Sample, n		Median survival, months		Univariate analysis		Multivariate analysis*	
	NIV	Non-NIV	NIV	Non-NIV	HR (95%CI)	P	HR (95%CI)	P
All phenotypes**	219	710	28.63	15.02	0.61 (0.51 to 0.73)	< 0.001	0.72 (0.60 to 0.88)	0.001
Bulbar ALS***	58	254	32.61	13.57	0.50 (0.36 to 0.70)	< 0.001	0.59 (0.41 to 0.83)	0.003

NIV = noninvasive ventilation; HR = hazard ratio; CI = confidence interval; ALS = amyotrophic lateral sclerosis;

* Multivariate models included the following variables: percutaneous endoscopic gastrostomy, riluzol, age at symptom onset, and sex;

** Analysis stratified according to phenotype and index year in the database (i.e., before or after 2003);

*** Analysis stratified according to index year in the database.

## DISCUSSION

It has been consistently demonstrated that NIV therapy relieves the symptoms of chronic hypoventilation and increases survival.^7^

Two studies evaluated the effects of NIV among patients with ALS and respiratory insufficiency with an indication for ventilatory support: a Cochrane systematic review and a retrospective analysis of a prospective cohort conducted in Australia. This systematic review included only a single eligible RCT with 41 participants; however, the methodological quality was considered adequate (low risk of bias).

As shown by the available evidence, patient survival is increased through the use of NIV, including in specific subgroups that are defined according to bulbar function^5^ and the ALS phenotype.^12^ In addition, the Cochrane systematic review conducted by Radunovic et al.^5^ demonstrated an improvement in quality-of-life parameters among patients who were treated with NIV for a longer time. The author identified scores > 75% above baseline measurements in the mental and physical components of the SF-36 instrument and in the SAQLI quality-of-life measurement.

NIV can provide a better quality of life for individuals in their homes and close to their families. It also delays the indication for tracheostomy, reduces expenditure on hospitalization, and reduces the occupancy of intensive care unit beds.^13^

Patients should begin NIV at the time of their first signs and symptoms of hypoventilation. Vital capacity is one of most commonly used clinical parameters; a decline greater than 50% of predicted value is associated with decreased chance of survival. Recent studies have attempted to optimize protocols for initiating NIV. A recent study demonstrated that there was an improvement in survival when the use of NIV was started with a vital capacity < 80% of the expected value and by incorporating a device for mechanical assistance for coughing.^6^

## CONCLUSION

The benefits of NIV in patients with ALS have been demonstrated over the last two decades. It improves the outcome and can delay the indication for tracheostomy, reduce expenditure on hospitalization, and increase the occupancy of intensive care unit beds.

The information presented in this review can be used as a source of knowledge for physicians and researchers to aid public policy strategies.

## NONINVASIVE MECHANICAL VENTILATION ASSISTANCE IN AMYOTROPHIC LATERAL SCLEROSIS: A SYSTEMATIC REVIEW

The Sao Paulo Medical Journal thanks Giulliano Gardenghi and the anonymous reviewer for their contributions to the peer review of this manuscript.

**Peer review reports**

**Table:**

Reviewer 1: Giulliano Gardenghi - ENCORE Hospital, Scientific Coordination
First evaluation
Recommendation: Accept
Comments: I consider this manuscript suitable for publication. Congratulations to the authors for the quality of the work. Reading the material was pleasurable and made me learn more about the subject.
Additional Questions: Does the manuscript contain new and significant information to justify publication? Yes Does the Abstract (Summary) clearly and accurately describe the content of the article? Yes Is the problem significant and concisely stated? Yes Are the methods described comprehensively? Yes Are the interpretations and conclusions justified by the results? Yes Is adequate reference made to other work in the field? Yes Is the language acceptable? Yes
Please rate the priority for publishing this article (1 is the highest priority, 10 is the lowest priority): 10.
Length of article is: Adequate. Number of tables is: Adequate. Number of figures is: Adequate.
Please state any conflict(s) of interest that you have in relation to the review of this paper (state “none” if this is not applicable): none
Rating: Interest: 1. Excellent. Quality: 1. Excellent. Originality: 1. Excellent. Overall: 1. Excellent.
Second evaluation
Recommendation: Accept
Comments: This manuscript, in my opinion is suitable for publication in its present form.
Additional Questions: Does the manuscript contain new and significant information to justify publication? Yes Does the Abstract (Summary) clearly and accurately describe the content of the article? Yes Is the problem significant and concisely stated? Yes Are the methods described comprehensively? Yes Are the interpretations and conclusions justified by the results? Yes Is adequate reference made to other work in the field? Yes Is the language acceptable? Yes
Please rate the priority for publishing this article (1 is the highest priority, 10 is the lowest priority): 2.
Length of article is: Adequate. Number of tables is: Adequate. Number of figures is: Adequate.
Please state any conflict(s) of interest that you have in relation to the review of this paper (state “none” if this is not applicable): none
Rating: Interest: 1. Excellent. Quality: 1. Excellent. Originality: 1. Excellent. Overall: 1. Excellent.
**Reviewer 2: Anonymous**
First evaluation
Recommendation: Minor Revision
Comments: Dear Authors, thank you for the opportunity to review your prominent paper that systematically reviewed the literature to call attention to the effectiveness and safety of noninvasive ventilation for patients with amyotrophic lateral sclerosis in respiratory failure. In addition, using the PICO strategy, the impact of noninvasive ventilation on survival and quality of the life in these patients was also evaluated. The paper reviewed has good originality, great scientific potential, and empirical application. Your group have identified the current panorama of the survey problem and directly responded to the questions raised. Finally, the information presented in the systematic review will possibly be used to guide physicians and researchers to develop and improve public policies, and to correct assessment and intervention measures. On the other hand, your paper needs adjustments for improving its quality. All criticisms were presented below. I. The manuscript demands corrections for grammar, spelling, and punctuation. I recommend an English proofreading service. Authors’ response: We agreed with the need for adjustments and provided grammar, spelling and punctuation modifications throughout the manuscript. II. Figures did not attend the PRISMA statement. II. 1. Selection and eligibility titles in the figure 1 were presented in small caps. The alignment of these two titles and of the term “Included” are not correct in relation to the text boxes. Authors’ response: We appreciate the comments and have made corrections to the titles and layout of the figures. II. 2. Figure 2 shows two lines without text boxes at the top. Authors’ response: Thank you for the feedback. We corrected the layout of the figure and text boxes. III. The two tables presented in the text are long. This condition difficult the interpretation. Authors’ response: We agreed and remade the tables, as can be found in the new paper submission. IV. The conclusion of the paper needs to be revisited. According to the PRISMA statement, this section provides a general interpretation of the results in the context of other evidence and implications for future research. In this sense, the conclusion presented in the abstract is more appropriate to attend to the statement. Please review the conclusion. Authors’ response: We appreciate the comments and have made modifications to the conclusion of the paper according to the suggestions. Additional Questions: Does the manuscript contain new and significant information to justify publication? Yes Does the Abstract (Summary) clearly and accurately describe the content of the article? Yes Is the problem significant and concisely stated? Yes Are the methods described comprehensively? Yes Are the interpretations and conclusions justified by the results? No Is adequate reference made to other work in the field? Yes Is the language acceptable? No
Please rate the priority for publishing this article (1 is the highest priority, 10 is the lowest priority): 2.
Length of article is: Adequate. Number of tables is: Adequate. Number of figures is: Adequate.
Please state any conflict(s) of interest that you have in relation to the review of this paper (state “none” if this is not applicable): None.
Rating: Interest: 1. Excellent. Quality: 2. Good. Originality: 2. Good. Overall: 2. Good.
**Second evaluation**
Recommendation: Accept
Comments: Congratulations to the authors for the great work.
I have no further requests or comments.
Additional Questions: Does the manuscript contain new and significant information to justify publication? Yes Does the Abstract (Summary) clearly and accurately describe the content of the article? Yes Is the problem significant and concisely stated? Yes Are the methods described comprehensively? Yes Are the interpretations and conclusions justified by the results? Yes Is adequate reference made to other work in the field? Yes Is the language acceptable? Yes
Please rate the priority for publishing this article (1 is the highest priority, 10 is the lowest priority): 2.
Length of article is: Adequate. Number of tables is: Adequate. Number of figures is: Adequate.
Please state any conflict(s) of interest that you have in relation to the review of this paper (state “none” if this is not applicable).: None.
Rating: Interest: 1. Excellent. Quality: 1. Excellent. Originality: 2. Good. Overall: 1. Excellent.
